# Effect of Prenatal Vitamin D and Selenium Supplementation on Minipuberty in Male Offspring of Women with Autoimmune Thyroiditis

**DOI:** 10.3390/nu18121993

**Published:** 2026-06-19

**Authors:** Karolina Kowalcze, Joanna Kula-Gradzik, Giuseppe Gullo, Simone Ferrero, Vito Chiantera, Robert Krysiak

**Affiliations:** 1 Department of Pediatrics in Bytom, Faculty of Health Sciences in Katowice, Medical University of Silesia, Stefana Batorego 15, 41-902 Bytom, Poland; jkula-gradzik@sum.edu.pl; 2 Department of Pathophysiology, Faculty of Medicine, Academy of Silesia, Rolna 43, 40-555 Katowice, Poland; 3 Department of Obstetrics and Gynecology, Villa Sofia Cervello Hospital, University of Palermo, 90146 Palermo, Italy; gullogiuseppe@libero.it; 4 Academic Unit of Obstetrics and Gynecology, IRCCS Ospedale Policlinico San Martino, 16132 Genoa, Italy; simone.ferrero@unige.it; 5 Department of Neurosciences, Rehabilitation, Ophthalmology, Genetics, Maternal and Child Health (DiNOGMI), University of Genoa, 16132 Genoa, Italy; 6 Department of Woman, Child and General and Specialized Surgery, University of Campania “Luigi Vanvitelli”, 80-138 Naples, Italy; vito.chiantera@unicampania.it; 7 Department of Internal Medicine and Clinical Pharmacology, Medical University of Silesia, Medyków 18, 40-752 Katowice, Poland; rkrysiak@sum.edu.pl

**Keywords:** genital organs, hypothalamic–pituitary–testicular axis, infants, pregnancy complications, saliva, thyroid autoimmunity

## Abstract

*Background/Objectives*: Minipuberty represents the second phase of physiological activation of the reproductive axis and may play a role in postnatal genital development. Its course has been shown to be affected by untreated or inadequately treated maternal hypothyroidism. The aim of the present study was to investigate minipuberty in the sons of women with euthyroid autoimmune thyroiditis during pregnancy. *Methods*: This prospective matched cohort study included three groups of apparently healthy infant boys. Two groups comprised the male offspring of levothyroxine-naive, euthyroid women with autoimmune thyroiditis: one group was unsupplemented, and the other received vitamin D and selenium supplementation. The control group consisted of boys born to healthy women. Salivary concentrations of testosterone, androstenedione, DHEA-S, estradiol, and progesterone, along with urinary FSH and LH levels, were assessed longitudinally over the first 12 months of life. These hormonal measurements were evaluated in relation to genital development, including testicular volume and penile length, which were recorded at each study visit. *Results*: Compared with the offspring of healthy mothers, sons of women with autoimmune thyroiditis who did not receive supplementation exhibited lower concentrations of LH and testosterone, without a distinct peak, while the duration of hormone detectability did not differ between the groups. These hormonal alterations were accompanied by reduced penile length, with no differences observed in testicular volume. This group also exhibited lower DHEA-S concentrations, whereas levels of other hormones were comparable. In contrast, in the group receiving vitamin D and selenium supplementation, the dynamics of hormonal changes and genital organ growth did not differ from those observed in the control group. LH concentrations were inversely correlated with thyroid peroxidase antibody titers, which were lower in the supplemented group. *Conclusions*: The findings indicate an altered course of minipuberty in the sons of women with euthyroid autoimmune thyroiditis during pregnancy and suggest a potential benefit of exogenous vitamin D and selenium supplementation in this population.

## 1. Introduction

Minipuberty refers to a temporary, sex-specific activation of the hypothalamic–pituitary–gonadal axis that occurs shortly after birth [1]. From a developmental standpoint, it represents the second activation of this axis, occurring after its prenatal activation in mid-gestation and before the pubertal resurgence in late childhood that enables sexual reproduction [2]. In healthy male infants, circulating levels of gonadotropins and testosterone gradually rise, reaching peak concentrations between 1 and 3 months of age, and subsequently decline to prepubertal levels between 6 and 9 months of age for follicle-stimulating hormone (FSH) and luteinizing hormone (LH), or by approximately 6 months of age for testosterone [2,3]. Activation of the reproductive axis during this period is associated with growth of the testes and penis, as well as changes in testicular cellular composition, including a transient increase in Leydig cell numbers, quantitative and morphological changes in immature Sertoli cells, and the differentiation of gonocytes into dark spermatogonia [4,5]. The sex-specific trajectory of minipuberty may also contribute to sex-related differences in growth velocity and fat distribution [6,7]. Finally, early activation of the reproductive axis may play a role in structural and functional brain development, influencing brain plasticity, gender identity, and the development of language and emotional competencies [4,8].

Findings from investigations carried out at our institution in recent years have demonstrated that uncorrected or insufficiently corrected maternal hormonal and metabolic disturbances during pregnancy—including hypothyroidism, gestational diabetes mellitus, and vitamin D deficiency—were linked to an atypical pattern of male minipuberty [9,10,11]. However, the sons of mothers affected by these conditions differed with respect to the nature of the observed changes, the timing of hormone detectability, and the presence or absence of peak hormone concentrations. These findings suggest that an altered pattern of minipuberty does not represent a nonspecific response to an adverse intrauterine environment independent of the underlying cause; rather, it reflects a distinct and condition-specific response unique to each disorder examined [9,10,11]. In male offspring of mothers with hypothyroidism, lower concentrations of gonadotropins and testosterone were observed during the first six months of life, accompanied by reduced penile length and smaller testicular volume compared with sons of healthy mothers [9]. These findings align with prior experimental evidence showing that maternal hypothyroidism induced during pregnancy led to both structural and quantitative testicular abnormalities in neonatal rodents, [12,13] as well as diminished FSH and LH concentrations and delayed testicular maturation at puberty [14]. In contrast, no abnormalities in the course of minipuberty were observed when maternal hypothyroidism was effectively treated during pregnancy [9,15].

It is likely that hypothyroidism is not the sole maternal thyroid condition associated with an increased risk of abnormal male minipuberty. This is supported by observations that sons of women with hyperthyroidism during pregnancy were characterized by an earlier onset and faster progression of puberty compared with sons of healthy mothers, and a similar—though not statistically significant—trend was noted in male offspring of women with mild nodular goiter [16]. To date, no study has specifically focused on evaluating the course of male minipuberty in the offspring of mothers with autoimmune thyroiditis. This disorder, commonly referred to as Hashimoto’s thyroiditis, is regarded as the most common thyroid disease in iodine-sufficient regions, the most prevalent autoimmune condition, and one of the most widespread human diseases overall; it affects women four to ten times more frequently than men [17,18]. Several considerations underscore the rationale for undertaking such studies. First, Hashimoto’s thyroiditis is often diagnosed in women during their reproductive years [17]. Second, outside areas of inadequate iodine intake, it constitutes the principal cause of hypothyroidism [18]. In addition, even when biochemical euthyroidism is present, individuals with Hashimoto’s thyroiditis frequently experience symptoms characteristic of thyroid hormone deficiency, suggesting impaired hormone availability at the tissue level [19]. Third, vitamin D insufficiency has been identified as a factor predisposing to the development of autoimmune thyroiditis [20]. When present during pregnancy, maternal vitamin D deficiency has been associated with higher gonadotropin and testosterone concentrations, as well as larger testicular and penile dimensions, in male offspring during the first half of the first year of life [11]. Fourth, the presence of autoimmune thyroiditis during pregnancy is linked to an increased risk of preterm delivery, low birth weight, and delivery of infants small for gestational age [21,22]. This is particularly pertinent because both prematurity and small-for-gestational-age status have been linked to alterations in the course of male minipuberty [23,24,25]. Furthermore, observational data indicate that maternal Hashimoto’s disease, even in the absence of overt thyroid dysfunction, may interfere with early neuropsychological development in offspring [26], a process influenced by the hormonal dynamics of the minipuberty period [8]. Recently, we have demonstrated that the presence of Hashimoto’s disease during gestation is associated with reduced activity of the hypothalamic–pituitary–gonadal axis in female offspring [27]. However, fundamental sex-based differences in the duration and hormonal dynamics characterizing this developmental period [2,3], as well as the poorly understood role of female mini-puberty, preclude drawing conclusions regarding boys. Therefore, the aim of the present study was to assess the course of minipuberty in sons of mothers with Hashimoto’s disease who remained euthyroid during pregnancy and, due to the absence of clinical indications [28], did not receive levothyroxine replacement therapy.

## 2. Materials and Methods

The study, designed as a prospective, matched cohort investigation, was conducted from April 2022 to October 2024. Approval for the research protocol was granted by the Bioethical Committee of the Medical University of Silesia (approval number: PCN/CBN/0052/KB1/17/22, approval date: 12 April 2022), and all procedures adhered to the ethical standards of the 1964 Declaration of Helsinki. The investigator fully informed the parents or legal guardians about the study’s objectives and potential risks, after which written informed consent was obtained on behalf of the child.

### 2.1. Participants

A total of 90 male infants born within the 30 days preceding study enrollment participated in the study. Recruitment targeted the population residing in the Silesian Voivodeship, located in southern Poland. Eligible children were referred to the study clinic by physicians collaborating with the research team, primarily neonatologists and pediatricians. The study protocol was designed to establish three groups, each comprising 30 participants. Groups 1 and 2 consisted of offspring of mothers with a pre-pregnancy diagnosis of Hashimoto’s disease who did not receive exogenous thyroid hormone therapy due to maintained euthyroidism. Group 3, serving as the control group, included offspring of healthy women. The reported group sizes exceeded the numbers calculated in an a priori sample size analysis performed before patient recruitment. This analysis determined that, to detect a 25% difference in salivary testosterone levels—the primary endpoint of the study—assuming a 5% type I error rate and a statistical power of 0.8, 25 participants per group were required. The inclusion of additional participants accounted for potential premature withdrawal from the study. To minimize the impact of seasonal variation on hormone secretion, participants were recruited in similar proportions across all four seasons.

Eligibility for groups 1 and 2 required the presence of thyroid peroxidase antibody (TPOAb) titers at least three times above the upper limit of normal, a characteristic ultrasonographic pattern of Hashimoto’s disease during pregnancy, and normal TSH levels—and, if measured, normal free thyroid hormone levels. At least two measurements of TPOAb were required, including at least one obtained within the 12 months preceding pregnancy and at least one obtained during pregnancy. Participants were also required to have at least four TSH measurements within the reference range, comprising one measurement taken within six months prior to pregnancy and three obtained during pregnancy, including at least one in the first trimester. Euthyroidism during pregnancy was defined according to the reference range recommended by the American Thyroid Association [28]. Participants were assigned to group 1 if they did not receive vitamin D or selenium supplementation during pregnancy, and to group 2 if they received daily doses exceeding 37.5 µg of vitamin D and 55 µg of selenium for at least six months during pregnancy. Patients receiving lower doses or supplementation for shorter periods were excluded from the study. Mothers of boys assigned to group 3 were required to have TSH levels within the normal range—defined as the reference range with the lower limit reduced by 0.4 mU/L in the first trimester and an upper limit of 4.0 mU/L—on at least two measurements during the 12 months preceding enrollment, including at least one measurement conducted during pregnancy. Additionally, they were required to have normal thyroid antibody titers during the six months preceding pregnancy or during pregnancy, as well as a thyroid gland of normal size and echogenicity during pregnancy (if assessed) and at the time of recruitment.

Maternal exclusion criteria included discordant laboratory results during pregnancy, isolated hypothyroxinemia (defined as free thyroxine concentrations below 10.0 pmol/L with normal TSH levels), any chronic medical condition, or pharmacological treatment during pregnancy or lactation (in breastfeeding women) for more than 10 days, with the exception of vitamin or micronutrient supplementation intended for pregnant or breastfeeding women. Maternal exclusion criteria also included pregnancy complications necessitating urgent hospitalization and a history of drug or alcohol abuse. Lastly, infants were excluded if they were diagnosed with genetic syndromes, major congenital malformations, cryptorchidism, congenital infections, or birth asphyxia; if they had any chronic disease; if they were born before 36 weeks of gestation; or if they required long-term pharmacological treatment, except for vitamin D supplementation.

**Figure 1:**
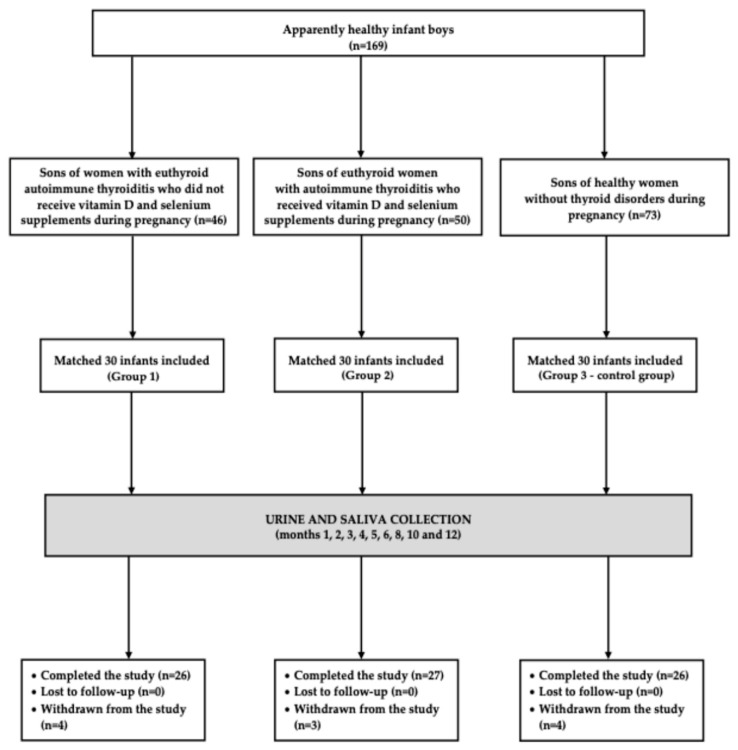
The progression of participants throughout the study.

Participants were drawn from a larger cohort of male infants who met the predefined inclusion and exclusion criteria (Figure 1). The selection process was designed to create three groups comparable with respect to maternal age, gestational age at delivery, birth order, and mean maternal TSH levels during pregnancy. Groups 1 and 2 were additionally matched for maternal TPOAb titers within the 12 months preceding pregnancy. All relevant maternal and neonatal data were obtained from medical records.

### 2.2. Calculated Parameters Reflecting Maternal Thyroid Homeostasis

To obtain a more comprehensive characterization of maternal thyroid homeostasis, calculated parameters derived from conventional thyroid function tests were assessed. These included Jostel’s TSH Index, which reflects the set point and responsiveness of the pituitary–thyroid feedback loop; SPINA-GT, which estimates the thyroid gland’s secretory output under conditions of maximal stimulation; and SPINA-GD, which reflects the efficiency of the peripheral conversion of thyroxine to the biologically active hormone triiodothyronine. Unlike isolated measurements of serum TSH and free thyroid hormone levels, these parameters provide functional insight into different components of the hypothalamic–pituitary–thyroid axis regulation [29,30]. Consequently, they may facilitate the detection of subtle alterations in thyroid function and improve the assessment of thyroid homeostasis in both clinical and research settings. Jostel’s TSH Index was calculated according to the formula: ln(TSH) + 0.1345 × free thyroxine. SPINA-GT was estimated using the following equation: SPINA-GT = βT × (DT + TSH) × (1 + K41 × [TBG] + K42 × [TTR]) × free thyroxine/(αT × TSH). SPINA-GD was calculated using the equation: SPINA-GD = β31 × (KM1 + free thyroxine) × (1 + K30 × [TBG]) × fT3/(α31 × free triiodothyronine), where [TBG] denotes the standard concentration of thyroxine-binding globulin and [TTR] denotes the standard concentration of transthyretin. The following model constants were used for all calculations: βT = 1.1 × 10^−6^ s^−1^, DT = 2.75 mU/L, K41 = 2 × 10^10^ L/mol, [TBG] = 300 nmol/L, K42 = 2 × 10^8^ L/mol, [TTR] = 4.5 μmol/L, αT = 0.1 L^−1^, β31 = 8 × 10^−6^ s^−1^, KM1 = 5 × 10^−7^ mol/L, K30 = 2 × 10^9^ L/mol, and α31 = 0.026 L^−1^ [29,31].

### 2.3. Study Design

The observation period for each patient during the study was 12 months. During the first six months, visits were conducted on a monthly basis, whereas in the second half of the first year of life, they took place at two-month intervals. Each visit began with the collection of a medical history from a parent or legal guardian concerning the infant’s development, their perception of the child’s health status, as well as any past illnesses, potential injuries or hospitalizations, and medications used since the previous visit. Subsequently, the investigator performed a physical examination and—if applicable—reviewed the results of laboratory and imaging studies. If, based on the overall assessment, the investigator determined that the infant was healthy and had not received any treatment within the preceding 10 days (with the exception of mandatory vaccinations), anthropometric measurements were obtained, and saliva and urine samples were collected for analysis. The final analysis included only data from infants for whom biological material had been collected at least seven times, including at least five collections during the first six months of life and at least two collections during the second half of the first year of life.

Vitamin D, selenium, energy, and macronutrient intake during pregnancy were evaluated at the initial study visit. For mothers who maintained dietary records throughout pregnancy, intake estimates were derived from analyses of those records. The remaining mothers completed a questionnaire assessing the quantity and frequency of consumption of commonly consumed Polish meals during the final eight weeks of pregnancy, with frequency categories defined as daily, 5–6 times per week, 3–4 times per week, 1–2 times per week, less frequently, or never. Mean daily dietary intake of both micronutrients was calculated based on the types, composition, and portion sizes of the reported foods, using standardized food composition and nutrition tables [32]. The dietary assessment scheme used in the study resulted from the nature of the research design. Although the follow-up of infants was prospective, the first contact between the research team and the mothers took place only after delivery. Since food records were completed during pregnancy by only some of the mothers, obtaining the necessary information from the remaining women required retrospective assessment. Limiting the questionnaire-based assessment to the last eight months of pregnancy increased the reliability of the obtained results. The applied study design, which included two different methods of dietary assessment, despite differences in accuracy, was considered more appropriate than relying exclusively on questionnaire-based analysis in all women [33].

All mothers, regardless of study group, were advised to maintain a daily intake of at least 37.5 µg of vitamin D and 55 µg of selenium throughout the lactation period. This recommendation was associated with the increased demand for both micronutrients during breastfeeding and, in women with autoimmune thyroiditis, with the frequent postpartum exacerbation of the disease [18]. Vitamin D content in breast milk was estimated indirectly using total milk consumption and the concentration of vitamin D in human milk. To estimate vitamin D concentration in breast milk, a non-linear lactational excretion profile was incorporated as a function of maternal daily intake, yielding predefined mean values of 1.0 μg/L for intakes below 25 μg, 2.5 μg/L for intakes between 25 and 50 μg, 3.5 μg/L for intakes between 50 and 160 μg, and 11.3 μg/L for intakes exceeding 160 μg [34,35]. To estimate selenium content in breast milk, maternal daily intake was multiplied by a constant of 0.175. This constant was derived from established coefficients, including gastrointestinal absorption efficiency of dietary selenium (0.7), fractional transfer of selenium into breast milk (0.2), and mean daily milk production (800 mL) [36]. For infants fed exclusively with infant formula, daily vitamin D and selenium intake was calculated based on their concentrations in the formula and the volume consumed. For infants receiving both breast milk and infant formula, intakes from both sources were combined. Furthermore, for vitamin D, recommended supplementation in the form of drops or twist-off capsules was taken into account.

### 2.4. Anthropometric Measurements

Body length was assessed from the vertex of the head to the plantar surface of the foot, with both lower limbs fully extended and the chin positioned perpendicular to the measuring surface. Measurements were recorded to the nearest 1 mm using a portable infantometer. Body weight was measured with a digital infant scale (Seca 834, Hamburg, Germany), accurate to 10 g, after removal of all clothing and diapers. Head circumference was measured from the occipital prominence to the supraorbital margins using a flexible, non-stretch measuring tape. Weight-for-length was calculated by dividing body weight by length and subsequently expressed as percentiles based on the World Health Organization growth standards.

Penile length was evaluated with the subject positioned supine and the lower extremities flexed, using a standardized calibrated measuring tape. The measurement was taken from the pubic ramus to the distal margin of the glans penis, excluding preputial tissue. Before each measurement, the penis was uniformly stretched to the point of maximal, non-painful resistance. All measurements were obtained in triplicate by the principal investigator, and the arithmetic mean of the three values was recorded as the final measurement.

Testicular volume was assessed using a high-resolution small-part transducer with a frequency range of 5–12 MHz. For each testis, separate longitudinal and transverse images were obtained, with the left testicle evaluated first. The longitudinal diameter (length) was obtained from the maximal longitudinal view, the anterior–posterior diameter (width) from the same plane, and the lateral-medial diameter (thickness) from the maximal transverse view. Volumes were calculated using Lambert’s equation (length × width × height × 0.71), which has demonstrated superior accuracy compared to other sonographic formulas. The epididymis was excluded from the volume assessment. Mean testicular volume was determined by summing the volumes of both testes and dividing by two. Each measurement was performed in triplicate, and the final value was recorded as the average of these three independent measurements.

### 2.5. Laboratory Assays

Biological material for biochemical analyses (urine and saliva) was collected between 7:30 and 9:30 a.m. in a separate room under conditions designed to ensure the child’s comfort. Urine samples were collected using sterile collection bags specifically designed for this purpose. After thoroughly washing the perineal and genital areas and subsequently drying the skin, the protective paper was removed from the adhesive, and the bag was affixed so that the opening surrounded the urethral meatus and the entire penis was positioned inside the bag. Once the bag was filled with urine, it was incised, and the contents were emptied into a dedicated collection container. Saliva samples were collected using sterile 2 mL syringes. The procedure involved placing the syringe into the oral cavity and aspirating saliva. Due to the noninvasive nature of the procedure, the high salivary output during the first year of life, and the experience of the person collecting the sample, the procedure was brief (lasting no longer than one minute), stress-free for the child, and well tolerated. To avoid contamination of the sample with food particles and the potential influence of feeding on the concentrations of the hormones being analyzed, saliva was collected no earlier than one hour after feeding. The collected samples were then centrifuged to remove epithelial cells and any other formed elements or contaminants. The resulting supernatant was transferred into polypropylene tubes. Urine and saliva samples were subsequently frozen and stored at −20 °C until analysis. To account for urine dilution, hormone concentrations were normalized to creatinine levels, which were determined using routine laboratory procedures in accordance with the method described by O’Brien et al. [37]. Salivary concentrations of testosterone, androstenedione, dehydroepiandrosterone sulfate (DHEA-S), and estradiol were assessed using enzyme-linked immunosorbent assay methods [9,11]. The analytical sensitivity (limits of detection [LOD]) of the assays was 0.1 U/L for both LH and FH, 10 pmol/L for testosterone, 18 pmol/L for androstenedione, 10 pmol/L for DHEA-S, 4 pmol/L for estradiol, and 16 pmol/L for progesterone.

### 2.6. Statistical Analysis

Results below the LOD were assigned the LOD value when at least one other result exceeded this threshold. When all results were below the LOD, statistical analysis was not performed. All quantitative data underwent logarithmic transformation to normalize the distribution prior to analysis. The study groups were compared using analysis of covariance (ANCOVA). Covariates included maternal smoking status, maternal body mass index, mean arterial blood pressure, average daily caloric intake during pregnancy, and breastfeeding status. Post hoc pairwise comparisons of adjusted means were conducted using the Bonferroni correction. Within-group changes over time were analyzed using repeated-measures analysis of variance (ANOVA), with pairwise comparisons adjusted using Tukey’s method. Comparisons of categorical variables were performed with the chi-square test. Pearson’s correlation was used for associations between two continuous variables. The phi coefficient was used for associations between two binary categorical variables. Point-biserial correlation was used for associations between a continuous variable and a binary categorical variable. Two-tailed *p*-values, after correction for multiple testing, less than 0.05 were considered statistically significant.

## 3. Results

Of the 90 boys initially enrolled, 79 (88%) completed the study. The most common reason for withdrawal, occurring in eight participants (two each from groups 1 and 2, and four from group 3), was failure to obtain the required number of samples due to intercurrent infections or parents’ inability to attend scheduled visits. One participant (group 1) discontinued due to atopic dermatitis requiring pharmacological treatment. In group 2, one boy withdrew after disclosure that his mother had Addison–Biermer anemia requiring long-term vitamin B_12_ therapy, including during pregnancy. In another case (group 1), parental consent was withdrawn for nonmedical family reasons. Post hoc power analysis confirmed that the number of participants who completed the study was sufficient for reliable statistical inference (Figure 1).

Comparisons between the mothers of male participants revealed no significant differences in age, educational attainment, employment status, type of occupation, parity, smoking habits, body mass index, or systolic and diastolic blood pressure between the study groups. Mean vitamin D and selenium intakes varied across groups, being highest in group B and similar in groups A and C (Table 1). The mean duration of supplementation during pregnancy was 32 ± 2 weeks. The study groups did not differ with respect to the dose or duration of vitamin D and selenium supplementation during lactation. Mean daily intakes of energy, carbohydrates, lipids, and proteins did not differ between the groups (Supplementary Table S1).

Before pregnancy, thyroid antibody titers did not differ between groups 1 and 2 and were higher in these groups than in group 3. No differences were observed in TSH, free thyroxine, SPINA-GT, and SPINA-GD between the study groups before pregnancy. Among women with autoimmune thyroiditis, TPOAb concentrations were higher before pregnancy than during pregnancy. Moreover, during pregnancy, TPOAb levels were lower in group 2 than in group 1. In neither group 1 nor group 2 were differences observed in TSH concentrations, free thyroid hormone levels, or Jostel’s index between the pre-pregnancy period and pregnancy. In group 2, SPINA-GT and SPINA-GD values increased during pregnancy compared with the pre-pregnancy period; this pattern was not observed in group 1. Additionally, during pregnancy, both parameters differed between the two groups (Table 2).

No significant differences were observed between the study groups in gestational age at delivery, birth order, body length, weight, weight-for-length percentile, head circumference, breastfeeding status, or total daily vitamin D and selenium intake (from both food and supplements) (Table 3).

**Figure 2:**
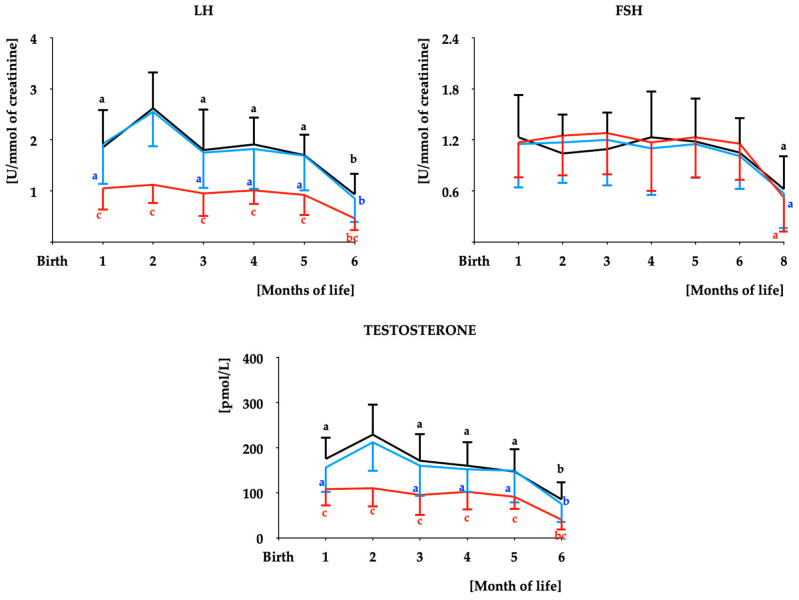
Urinary gonadotropin and salivary testosterone concentrations in infant boys participating in the study. Data are presented as mean ± standard deviation. Each subplot depicts only the periods during which the respective hormone was detected. Red line: group 1 (sons of women with euthyroid autoimmune thyroiditis who did not receive vitamin D and selenium supplementation during pregnancy); blue line: group 2 (sons of women with euthyroid autoimmune thyroiditis who received vitamin D and selenium supplementation during pregnancy); black line: group 3 (sons of healthy women without thyroid disorders during pregnancy). Statistical annotations: LH and testosterone, ᵃ *p* < 0.05 compared with levels at month 2 within the same study group; ᵇ *p* < 0.05 compared with levels at months 1–5 within the same study group; ᶜ *p* < 0.05 compared with the corresponding values at the same time point in the other study groups. FSH, ᵃ *p* < 0.05 compared with levels at months 1–6 within the same study group.

In all study groups, LH was detectable in urine during the first six months of life. Throughout this period, LH concentrations were lower in group 1 than in the other groups, with no differences observed between groups 2 and 3. No peak LH concentrations were observed in group 1, whereas in the other groups, the peak occurred in the second month of life. In all groups, LH concentrations at six months of age were lower than those observed at earlier time points. FSH was detectable in urine in all groups during the first eight months of life. Concentrations remained comparable across groups during the first six months and subsequently declined. No differences in FSH concentrations between groups were observed at any assessed time point. Testosterone was detectable in urine in all groups during the first six months of life. Throughout this period, concentrations were lower in group 1 than in the other groups, with no differences observed between groups 2 and 3. No peak testosterone concentrations were observed in group 1, whereas a peak was observed in the second month of life in the remaining groups. In all groups, testosterone concentrations at six months of age were lower than those observed at earlier time points (Figure 2).

**Figure 3:**
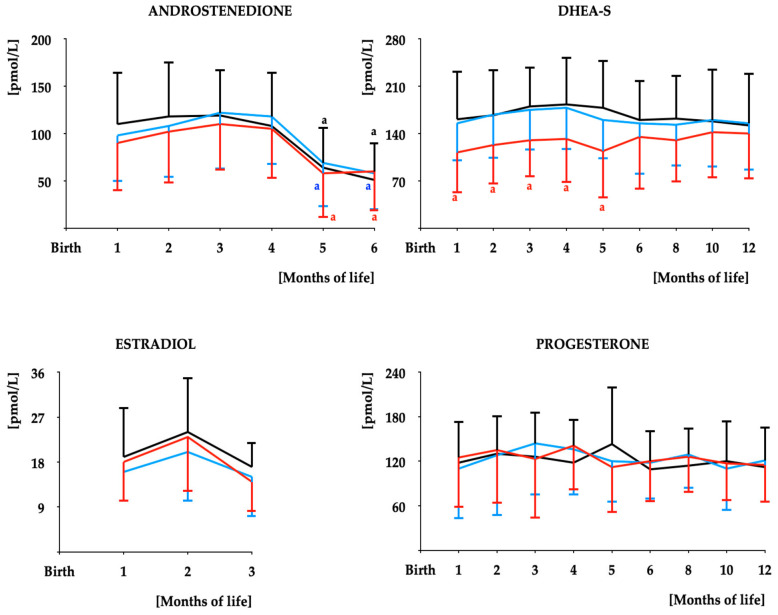
Salivary concentrations of other steroid hormones in infant boys participating in the study. Data are presented as mean ± standard deviation. Each subplot depicts only the periods during which the respective hormone was detected. Red line: group 1 (sons of women with euthyroid autoimmune thyroiditis who did not receive vitamin D and selenium supplementation during pregnancy); blue line: group 2 (sons of women with euthyroid autoimmune thyroiditis who received vitamin D and selenium supplementation during pregnancy); black line: group 3 (sons of healthy women without thyroid disorders during pregnancy). Statistical annotations: androstenedione, ᵃ *p* < 0.05 for months 1–4 compared with baseline values within the same study group; DHEA-S, ᵃ *p* < 0.05 compared with the corresponding values at the same time point in the other study groups.

Androstenedione was detectable in saliva during the first six months of life, with concentrations declining after the fourth month in all groups. No differences between groups were observed at corresponding time points. DHEA-S was detectable in saliva throughout the study period. No within-group differences were observed over the entire study. During the first five months of life, salivary DHEA-S concentrations were lower in group 1 than in the other groups. Estradiol was detectable in urine during the first three months of life. Concentrations remained comparable across groups and did not differ between them. Progesterone was detectable in saliva throughout the study period, with concentrations remaining comparable across groups (Figure 3).

**Figure 4:**
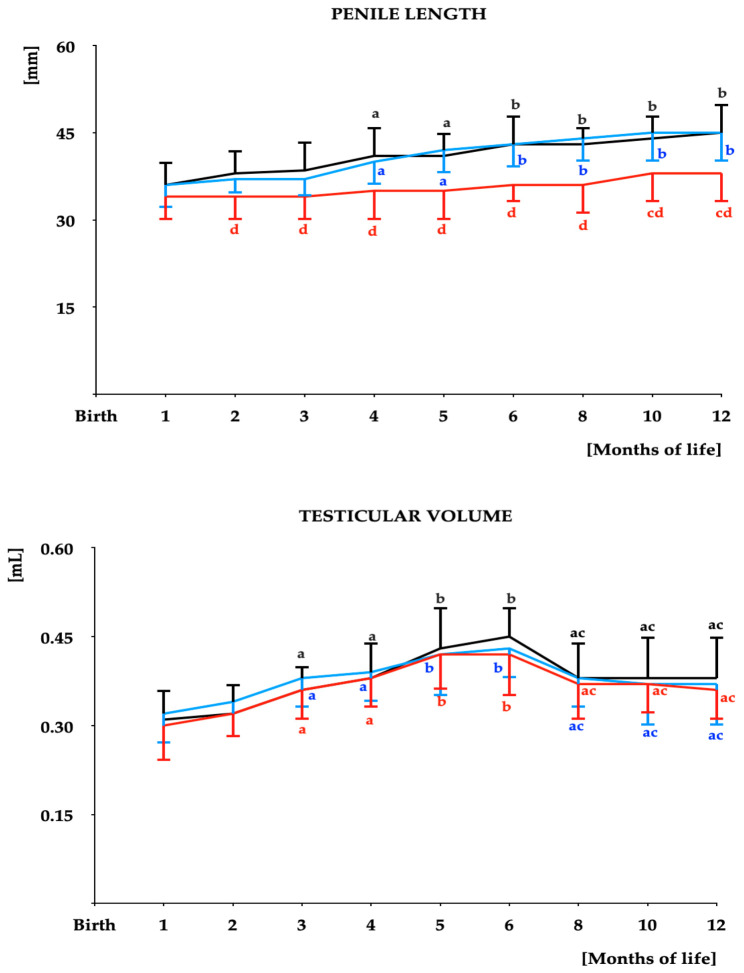
Penile length and testicular volume in infant boys participating in the study. Data are presented as mean ± standard deviation. Each subplot depicts only the periods during which the respective hormone was detected. Red line: group 1 (sons of women with euthyroid autoimmune thyroiditis who did not receive vitamin D and selenium supplementation during pregnancy); blue line: group 2 (sons of women with euthyroid autoimmune thyroiditis who received vitamin D and selenium supplementation during pregnancy); black line: group 3 (sons of healthy women without thyroid disorders during pregnancy). Statistical annotations: penile length, ᵃ *p* < 0.05 compared with values at months 1–3 within the same study group; ᵇ *p* < 0.05 compared with values at months 1–4 within the same study group; ᶜ *p* < 0.05 compared with values at months 1–8 within the same study group; ᵈ *p* < 0.05 compared with the corresponding values at the same time point in the other study groups. Testicular volume, ᵃ *p* < 0.05 compared with values at months 1–2 within the same study group; ᵇ *p* < 0.05 compared with values at months 3–4 within the same study group; ᶜ *p* < 0.05 compared with values at months 5–6 within the same study group.

In group 1, a statistically significant increase in penile length was observed from 10 months of age, whereas in the other groups, the increase was observed from four months of age. From the second month onward, penile length was smaller in group 1 than in the other groups, with no differences between groups 2 and 3. In all study groups, testicular volume increased from birth to five to six months of age, at which point it reached a maximum, followed by a subsequent decline. No differences between groups were observed at corresponding time points (Figure 4).

Throughout the study period, positive associations were observed in all groups between urinary LH and salivary testosterone, between salivary testosterone and penile length, and between urinary FSH and testicular volume. In group 1, there were inverse correlations between TPOAb titers and salivary DHEA-S concentrations. In groups 1 and 2, maternal TPOAb titers during pregnancy were inversely correlated with total vitamin D and selenium intake during pregnancy, salivary LH levels, and gestational SPINA-GT (Table 4).

## 4. Discussion

Due to the inclusion criteria applied in the study, all women with autoimmune thyroiditis remained euthyroid throughout pregnancy. Despite comparable pre-pregnancy antibody levels and their decline during pregnancy in both groups of mothers with Hashimoto’s disease, these levels were lower in those receiving vitamin D and selenium supplementation. These observations support a beneficial effect of combined vitamin D and selenium administration on thyroid autoimmunity in women of reproductive age [38], providing the first evidence of such an effect during pregnancy. Another notable finding was the effect of supplementation on SPINA-GT, which appears to be a more sensitive marker of thyroid secretory function than serum free hormone concentrations [29,30]. In turn, low SPINA-GD values appear to indicate impaired conversion of thyroxine to triiodothyronine [29] and likely reflect selenium deficiency, which is common in the study region [39]. This interpretation is supported by the observed increase in the parameter in the supplemented group, suggesting enhanced deiodinase activity in response to improved selenium homeostasis. Importantly, differences in SPINA-GT and SPINA-GD were observed despite their calculation being possible in only approximately half of the mothers with available free thyroid hormone measurements.

The most important finding of the present study was the demonstration of lower concentrations of testosterone and LH, as well as the absence of a peak in the concentrations of both hormones, in the sons of mothers with euthyroid Hashimoto’s disease who did not receive vitamin D and selenium supplementation, compared with the male offspring of healthy mothers, with no differences observed in the duration of detectability of both hormones (6 months). As a consequence of lower LH levels with FSH concentrations comparable to those observed in the control group, the sons of mothers with Hashimoto’s disease who did not receive supplementation exhibited higher mean FSH concentrations than LH concentrations, a pattern characteristic of physiological female minipuberty (whereas in boys, LH concentrations typically exceed FSH concentrations) [3]. The pattern observed in the male offspring of euthyroid women with untreated autoimmune thyroiditis differed significantly from that reported in male offspring of mothers with uncontrolled or inadequately controlled hypothyroidism during pregnancy. In the sons of the latter group, lower FSH concentrations were also observed [9]. Notably, an analogous pattern was observed during minipuberty in daughters of women with autoimmune thyroiditis, who exhibited lower LH and estradiol concentrations, whereas FSH levels remained unaffected [27].

The applied research protocol and the results obtained allow for several noteworthy conclusions. First, the use of stringent exclusion criteria ensures that the observed changes in the course of male minipuberty cannot be attributed to other maternal diseases, medication use, or pathological course of pregnancy or delivery-related complications. Second, the altered course of minipuberty cannot be explained by pregnancy or birth complications in the infant itself, nor by the presence of other pathological conditions in the offspring. Third, the impact of different thyroid disorders on the course of male minipuberty varies. This not only supports our hypothesis regarding the distinct effects of different pathological states on this phase of reproductive axis activation but also, for the first time, indicates that different disorders of the same organ exert different effects during this period. It is therefore warranted to conduct analogous studies in the context of other thyroid diseases. Fourth, the course of male minipuberty in the offspring of mothers with Hashimoto’s disease differs from that observed under physiological conditions, despite the maintenance of a euthyroid state during pregnancy. This suggests that the influence of this disorder on hormonal changes in the hypothalamic-pituitary-gonadal axis during the first year of life occurs even in the absence of alterations in hypothalamic-pituitary-thyroid axis activity. Accordingly, euthyroid Hashimoto’s disease during pregnancy should not be regarded as a benign condition without potential clinical consequences.

The presence of positive correlations between salivary testosterone and urinary LH concentrations, as well as identical periods of hormone detectability, suggests that the increased testicular hormonal activity is most likely secondary to altered pituitary LH secretion. The potential sites of influence of autoimmune thyroiditis may include gonadotropic cells and/or the hypothalamus, which produces the most potent stimulator of LH secretion—gonadotropin-releasing hormone [40]. The altered course of minipuberty does not appear to reflect differences, even subtle ones, in hypothalamic-pituitary-thyroid axis activity. Any abnormalities in TSH or free thyroid hormone concentrations led to exclusion from the study. Furthermore, even for parameters that differed between groups during pregnancy (SPINA-GT and SPINA-GD), no correlations were observed between these parameters and LH or testosterone concentrations (nor with the concentrations of other evaluated hormones) in the infant. Although the coexistence of maternal euthyroidism with subtle tissue-level thyroid hormone deficiency cannot be entirely excluded, our study does not provide evidence to support this explanation. These findings are consistent with the highest TPOAb titers during pregnancy observed in mothers with Hashimoto’s disease who did not receive supplementation, as well as the presence of negative correlations between TPOAb titers during pregnancy and LH concentrations in the child. Similar correlations were not assessed for TgAb, as their lower sensitivity and specificity [41] led to evaluation in a smaller proportion of patients and very infrequent measurements during pregnancy.

The study protocol does not determine whether the effect of maternal autoimmune thyroid disease on minipuberty is mediated by TPOAb (which can penetrate the placenta [42]), other autoimmune mediators, or systemic inflammation. However, two hypotheses may explain the delay between exposure to maternal autoimmune disease and altered activation of the reproductive axis in infancy. First, the autoimmune process may affect the initial intrauterine phase of gonadal axis activation, leading to later changes during minipuberty. Second, maternal thyroid disease may affect fetal programming by inducing epigenetic changes in the fetal genome, similar to other adverse environmental exposures [43]. This may result in altered hypothalamic-pituitary-gonadal axis activation in male offspring.

One of the most notable findings of the present study was that hormonal changes within the reproductive axis characteristic of the minipuberty period did not differ from those observed in the control group when pregnant women received supplements containing vitamin D and selenium at doses exceeding 37.5 µg and 55 µg, respectively. This finding supports the appropriateness of such supplementation in pregnant women with Hashimoto’s disease who do not require hormonal replacement therapy. Of note, no differences in the course of minipuberty were observed despite TPOAb titers during pregnancy remaining higher than those in the control group. This may suggest that disturbances of male minipuberty occur only once the autoimmune process reaches a certain threshold. Unfortunately, the study design does not permit assessment of the individual contribution of each micronutrient, and it cannot be excluded that the entire observed effect was driven by a single supplemented component, underscoring a key limitation of the study. However, a potential synergistic effect of vitamin D and selenium supplementation on the course of minipuberty may be suggested by the observed inverse correlations between maternal TPOAb titers and maternal intake of each of these micronutrients during pregnancy, as well as by evidence indicating that exogenous selenium augments the beneficial effect of vitamin D in attenuating thyroid autoimmunity in young nonpregnant women [38]. To conclusively address this question, a study comparing the course of minipuberty in the offspring of women who, during pregnancy, received supplementation with vitamin D alone, selenium alone, or both agents in combination would be warranted.

The results obtained suggest that altered reproductive axis activity in male offspring of euthyroid women with untreated Hashimoto’s disease leads to a distinct clinical phenotype characterized by reduced penile growth compared with sons of healthy mothers, without differences in testicular volume. These findings differ from those observed in sons of mothers with hypothyroidism, who had reduced penile and testicular dimensions [9]. In the present study, penile growth in sons of mothers with autoimmune thyroiditis became statistically significant only at 10 months of age; however, from the second month onward, penile length remained lower than in controls. It remains unclear whether these differences affect pubertal penile growth rates or final length. Penile length correlated with salivary testosterone concentration, indicating that reduced growth reflects decreased reproductive axis activity. This is consistent with prior reports that penile growth of approximately 1 mm per month in the first months of life serves as a clinical indicator of adequate testosterone action [44]. This interpretation is further supported by normal penile size and growth in cases receiving vitamin D and selenium supplementation, in whom testosterone dynamics were comparable to those of controls. In contrast, no between-group differences in testicular volume were observed. As previously reported [45], testicular volume peaked at 5–6 months of age and subsequently declined. In early childhood, Sertoli cell function is primarily regulated by FSH due to the absence of androgen receptors [3]; therefore, unchanged FSH dynamics in offspring of women with euthyroid Hashimoto’s disease likely explain the absence of differences in testicular volume trajectories.

Another notable finding was the presence of lower salivary DHEA-S concentrations during the first five months of life in sons of mothers with Hashimoto’s disease who did not receive vitamin D and selenium supplementation, whereas no such abnormalities were observed in the supplemented group. Similar alterations have previously been reported in sons of mothers with gestational diabetes mellitus [10]. DHEA-S, the predominant circulating form of DHEA, is present at substantially higher concentrations than its parent compound [46]. If low DHEA and DHEA-S levels are a nonspecific marker of poor health associated with systemic and metabolic disorders, these findings may reflect an abnormal infant response to chronic intrauterine alterations caused by maternal autoimmune disease [46]. Alternatively, maternal disease may impair the paracellular transport of DHEA-S, which, due to its hydrophilic nature, does not enter saliva by diffusion, unlike the other hormones assessed [47]. Lastly, despite unchanged androstenedione levels, reduced adrenal steroidogenesis or impaired DHEA sulfation cannot be ruled out. While the reason for low DHEA-S levels during minipuberty remains unclear, reduced concentrations in early infancy may have adverse effects, given the neuroprotective role of DHEA and its importance in central nervous system development and maturation [48].

The course of minipuberty in male offspring of mothers with Hashimoto’s disease was entirely different from that observed in sons of mothers with vitamin D deficiency during pregnancy, who were characterized by increased gonadal axis activity [11]. These infants exhibited elevated concentrations of gonadotropins and testosterone during the first six months of life, an earlier peak in LH and testosterone levels, as well as larger penile and testicular dimensions compared with offspring of mothers with adequate vitamin D status [11]. Therefore, the changes observed in the present study do not appear to result from concomitant vitamin D deficiency and the normalization of vitamin D homeostasis following supplementation. Additional findings further argue against this explanation. No statistically significant differences in vitamin D intake were observed between mothers with Hashimoto’s disease who did not receive supplementation and healthy mothers. Maternal vitamin D intake showed no direct correlation with the concentrations of the hormones evaluated or with the size of the organs examined; it was associated exclusively with TPOAb titers. Theoretically, the impact of supplementation on the course of minipuberty could be explained by an improvement in low selenium status [39]. However, this explanation is inconsistent with the absence of correlations between the hormone and organ outcome measures and selenium intake or SPINA-GD, which assesses the activity of selenium-containing deiodinases [29]. Finally, a potential effect of differences in vitamin D and selenium intake through breast milk due to additional maternal supplementation during lactation appears unlikely, given the comparable intake across study groups (for vitamin D, because of mandatory infant supplementation) and the statistical adjustment applied in the analysis.

Additional limitations of the study should be acknowledged. Although the current sample size satisfies statistical requirements, the inclusion of only 30 participants per group may limit the generalizability of the findings. An increased sample size would improve statistical power. Cohort and single-center studies are inherently susceptible to bias and confounding factors, which may compromise the validity and reliability of the findings [49]. Because participants were enrolled relatively late, in the second half of the first month, the study was unable to capture hormonal changes occurring during the earliest stage of minipuberty (the first few days after birth). Due to the limited specificity of the antibodies, the immunoassays employed in this study may overestimate steroid concentrations as a result of cross-reactivity with structurally related molecules [50]. The study did not assess maternal iodine status. However, owing to mandatory prophylaxis, Poland is classified as a country with adequate iodine intake [51]. It cannot be excluded that the impact of maternal autoimmune thyroiditis on minipuberty in male offspring may differ in regions with insufficient iodine supply. Although design-stage safeguards were applied, the findings may still be affected by regression dilution due to long-term physiological changes in the outcome variables and/or regression to the mean, in which extreme values naturally shift toward the population average over time [52,53]. Lastly, the study protocol does not allow for conclusions to be drawn regarding the cellular and molecular mechanisms underlying the observed differences in the course of minipuberty.

## 5. Conclusions

Sons of levothyroxine-naive, euthyroid women with Hashimoto’s disease who did not receive vitamin D or selenium supplementation during pregnancy showed lower LH and testosterone concentrations, as well as the absence of hormone peaks over the six-month observation period. Accordingly, these infants had a less pronounced increase in penile size compared with the sons of healthy mothers. With unchanged FSH dynamics, testicular growth remained consistent with physiological minipuberty. Untreated autoimmune thyroiditis was also associated with a transient reduction in salivary DHEA-S concentrations. No hormonal alterations were observed with vitamin D and selenium supplementation, although supplementation reduced, but did not normalize, TPOAb titers. The beneficial effect of combined micronutrient administration appears to stem from reduced thyroid autoimmunity rather than from effects on the maternal hypothalamic-pituitary-thyroid axis or normalization of vitamin D and selenium status. These findings suggest that euthyroid autoimmune thyroiditis may alter male minipuberty and support the potential use of vitamin D and selenium supplementation in affected women when levothyroxine is not indicated. Given the study’s novelty, potential clinical relevance, and methodological limitations, these results require confirmation in further research. Additional studies are needed to clarify the relationship between altered minipuberty, later sexual maturation, and long-term adult outcomes.

## Figures and Tables

**Table 1:** Characteristics of mothers of male infants enrolled in the study (the intent-to-treat analysis).

Variable	Group 1	Group 2	Group 3
Number (n)	30	30	30
Age (years)	30 ± 6	31 ± 8	31 ± 7
Primary or vocational/secondary/university education (%)	13/43/43	17/40/43	13/37/50
Occupational activity/white-collar/pink-collar/blue-collar workers (%)	87/27/43/17	90/37/43/20	90/30/40/20
Number of deliveries (n)	1.4 ± 0.5	1.5 ± 0.7	1.2 ± 0.6
Smokers: overall/daily/occasional (%)	20/7/13	20/7/13	17/7/10
Body mass index (kg/m^2^)	24.4 ± 3.8	24.2 ± 3.7	23.9 ± 2.9
Systolic blood pressure (mmHg)	120 ± 18	118 ± 16	117 ± 15
Diastolic blood pressure (mmHg)	81 ± 7	80 ± 7	80 ± 6
Total daily vitamin D intake (µg)	14.2 ± 3.9	63.4 ± 11.2 ^a^	21.1 ± 18.8
Total daily selenium intake (µg)	41 ± 12	123 ± 21 ^a^	52 ± 28

Unless otherwise indicated, data are reported as mean values with corresponding standard deviations. Group 1: Women with euthyroid autoimmune thyroiditis who did not receive vitamin D and selenium supplementation during pregnancy; Group 2: Euthyroid women with autoimmune thyroiditis who received vitamin D and selenium supplementation during pregnancy; Group 3: Healthy women without thyroid disorders during pregnancy. ^a^ *p* < 0.05 compared with the remaining groups.

**Table 2:** Thyroid antibodies, hormones, and calculated parameters of thyroid homeostasis in mothers of male infants enrolled in the study (the intent-to-treat analysis).

Variable	Group 1	Group 2	Group 3
TPOAb [U/mL]			
Before pregnancy	865 ± 320 ^b^ (n = 30)	920 ± 295 ^b^ (n = 30)	14 ± 10 (n = 28)
During pregnancy	590 ± 285 ^c^ (n = 30)	395 ± 185 ^a,c,d^ (n = 30)	12 ± 14 (n = 6)
TgAb [U/mL]			
Before pregnancy	843 ± 355 ^b^ (n = 26)	884 ± 364 ^b^ (n = 25)	16 ± 14 (n = 12)
During pregnancy	610 ± 288 (n = 8)	412 ± 255 ^c^ (n = 10)	not assessed
TSH [mU/L]			
Before pregnancy	2.2 ± 1.2 (n = 30)	2.1 ± 1.0 (n = 30)	1.6 ± 1.2 (n = 30)
During pregnancy	2.1 ± 1.1 (n = 30)	1.9 ± 0.9 (n = 30)	1.6 ± 1.1 (n = 30)
Free thyroxine [mU/L]			
Before pregnancy	13.9 ± 3.5 (n = 30)	13.8 ± 3.2 (n = 30)	14.2 ± 3.8 (n = 8)
During pregnancy	13.5 ± 2.9 (n = 21)	15.7 ± 4.6 (n = 23)	not assessed
Free triiodothyronine [pmol/L]			
Before pregnancy	3.2 ± 1.2 (n = 28)	3.1 ± 1.4 (n = 27)	not assessed
During pregnancy	3.1 ± 1.3 (n = 18)	3.9 ± 1.7 (n = 20)	not assessed
Jostel’s TSH index			
Before pregnancy	2.7 ± 0.5 (n = 30)	2.6 ± 0.4 (n = 30)	2.4 ± 0.6 (n = 8)
During pregnancy	2.6 ± 0.5 (n = 21)	2.8 ± 0.5 (n = 23)	not assessed
SPINA-GT [pmol/s]			
Before pregnancy	2.35 ± 0.90 (n = 30)	2.42 ± 0.80 (n = 30)	2.93 ± 1.01 (n = 8)
During pregnancy	2.37 ± 0.86 (n = 21)	2.92 ± 0.72 ^a,c,d^ (n = 23)	not assessed
SPINA-GD [nmol/s]			
Before pregnancy	21.42 ± 2.95 (n = 28)	20.77 ± 4.00 (n = 27)	not assessed
During pregnancy	20.96 ± 2.80 (n = 18)	22.97 ± 3.02 ^a,c,d^ (n = 20)	not assessed

Data are reported as mean values with corresponding standard deviations. The number of patients in each group at each time point is indicated in parentheses. Group 1: Sons of women with euthyroid autoimmune thyroiditis who did not receive vitamin D and selenium supplementation during pregnancy; Group 2: Sons of euthyroid women with autoimmune thyroiditis who received vitamin D and selenium supplementation during pregnancy; Group 3: Sons of healthy women without thyroid disorders during pregnancy. ^a^ *p* < 0.05 compared with group 1; ^b^ *p* < 0.05 compared with group 3; ^c^ *p* < 0.05 compared with values before pregnancy; ^d^ *p* < 0.05 indicates percentage differences compared with the pre-pregnancy period that are more pronounced than those observed in group 1.

**Table 3:** Initial characteristics of infant participants in the intent-to-treat analysis.

Variable	Group 1	Group 2	Group 3
Number (n)	30	30	30
Gestational age of delivery (weeks)	40 ± 1	39 ± 2	39 ± 2
Birth order: first/second/third and subsequent (%)	53/37/10	50/37/13	53/40/7
Length (cm)	54.7 ± 1.7	54.9 ± 1.9	54.2 ± 1.8
Weight (kg)	4.64 ± 0.55	4.58 ± 0.60	4.51 ± 0.53
Weight-for-length percentile	70 ± 15	65 ± 18	71 ± 14
Head circumference (cm)	37.1 ± 0.8	37.3 ± 0.9	37.4 ± 0.8
Breastfeeding (%)	83	80	77
Total daily vitamin D intake (µg)	12.8 ± 1.6	13.4 ± 1.8	13.2 ± 1.5
Total daily selenium intake (µg)	10.9 ± 1.2	11.2 ± 1.3	11.4 ± 1.2

Unless otherwise indicated, data are reported as mean values with corresponding standard deviations. Group 1: Sons of women with euthyroid autoimmune thyroiditis who did not receive vitamin D and selenium supplementation during pregnancy; Group 2: Sons of euthyroid women with autoimmune thyroiditis who received vitamin D and selenium supplementation during pregnancy; Group 3: Sons of healthy women without thyroid disorders during pregnancy.

**Table 4:** Correlations between the measured variables.

Correlated Variables		Group 1	Group 2	Group 3
LH	Testosterone	0.49 [*p* = 0.0001] −0.64 [*p* < 0.0001]	0.51 [*p* < 0.0001] −0.71 [*p* < 0.0001]	0.53 [*p* < 0.0001] −0.74 [*p* < 0.0001]
Gestational TPOAb	Total vitamin D intake during pregnancy	−0.46 [*p* = 0.0001]	−0.48 [*p* < 0.0001]	not assessed
Gestational TPOAb	Total selenium intake during pregnancy	−0.43 [*p* = 0.0008]	−0.42 [*p* = 0.0009]	
Gestational TPOAb	LH	−0.41 [*p* = 0.0012]–−0.60 [ *p* < 0.0001]	−0.43 [*p* = 0.0009]–−0.58 [*p* < 0.0001]	not assessed
Gestational TPOAb	DHEA-S	−0.28 [*p* = 0.0468]–−0.38 [ *p* = 0.0206]	−0.04 [*p* = 0.4231]–−0.18 [*p* = 0.0908]	not assessed
Gestational TPOAb	Gestational SPINA-GT	−0.30 [*p* = 0.0401]	−0.42 [*p* = 0.0008]	not assessed
Testosterone	Penile length	0.39 [*p* = 0.0016] −0.50 [*p* < 0.0001]	0.46 [*p* = 0.0008] −0.58 [*p* < 0.0001]	0.41 [*p* = 0.0010] −0.56 [*p* < 0.0001]
FSH	Testicular volume	0.29 [*p* = 0.0424] −0.38 [*p* = 0.0128]	0.31 [*p* = 0.0382] −0.46 [*p* = 0.0001]	0.34 [*p* = 0.0235] −0.48 [*p* = 0.0001]

The data represent the correlation coefficients (r values). Group 1: Sons of women with euthyroid autoimmune thyroiditis who did not receive vitamin D and selenium supplementation during pregnancy; Group 2: Sons of euthyroid women with autoimmune thyroiditis who received vitamin D and selenium supplementation during pregnancy; Group 3: Sons of healthy women without thyroid disorders during pregnancy.

## Data Availability

The data presented in this study are available on request from the corresponding author due to privacy concerns.

## Supplementary Materials

The following supporting information can be downloaded at: https://www.mdpi.com/article/10.3390/nu18121993/s1, Table S1. Mean daily energy and macronutrient intake during pregnancy by mothers of male infants enrolled in the study (the intent-to-treat analysis).
