# Supplementary material for: Effect of Prenatal Vitamin D and Selenium Supplementation on Minipuberty in Male Offspring of Women with Autoimmune Thyroiditis
Source: Nutrients. 2026 Jun 19;18(12):1993. doi: 10.3390/nu18121993 (PMC13306070; doi:10.3390/nu18121993)
Supplement: Supplementary file 1 [file nutrients-18-01993-s001.zip › nutrients-4351351-supplementary.pdf]

Supplementary Table S1. Mean daily energy and macronutrient intake during pregnancy by mothers of male infants enrolled in the study (the intent-to-treat analysis).

Variable	Group 1	Group 2	Group 3
Dietary records/questionnaires (%)	50/50	43/57	47/53
Mean total daily calorie intake (kcal/kg)	29.5 ± 3.7	30.8 ± 4.1	29.8 ± 4.0
Mean daily carbohydrate intake (g)	306 ± 48	295± 52	283± 55
Mean daily lipid intake (g)	91 ± 11	93 ± 12	86 ± 15
Mean daily protein intake (g)	87 ± 16	92 ± 11	90 ± 14

Data are reported as mean values with corresponding standard deviations. Group 1: Women with euthyroid autoimmune thyroiditis who did not receive vitamin D and selenium supplementation during pregnancy; Group 2: Euthyroid women with autoimmune thyroiditis who received vitamin D and selenium supplementation during pregnancy; Group 3: Healthy women without thyroid disorders during pregnancy.
